# Exploration of immobilization conditions of cellulosic lyotropic liquid crystals in monomeric solvents by *in situ* polymerization and achievement of dual mechanochromism at room temperature†

**DOI:** 10.1039/c8ra04878a

**Published:** 2018-07-10

**Authors:** K. Miyagi, Y. Teramoto

**Affiliations:** Department of Applied Life Science, Faculty of Applied Biological Sciences, Gifu University Gifu 501-1193 Japan teramoto@gifu-u.ac.jp; Center for Highly Advanced Integration of Nano and Life Sciences (G-CHAIN), Gifu University Gifu 501-1193 Japan

## Abstract

We investigated conditions to prepare cellulosic cholesteric liquid crystalline (ChLC) films in order to accomplish dual mechanochromism, *i.e.*, colour control and circular dichroic inversion upon mechanical stimulus, at room temperature. Flexible propionylated hydroxypropyl cellulose (PHPC) was prepared by a simple reaction and found to be capable of forming lyotropic ChLC in various monomeric solvents. The ChLC solutions were subjected to *in situ* polymerization to obtain PHPC/synthetic polymer composite films incorporating the ChLC structure. However, the immobilization behaviour depended on the type of original monomers. Differential scanning calorimetry and solid-state NMR measurement revealed that the ChLC structure was more highly fixed when the compatibility between PHPC and the coexisting polymers was lower. Eventually, thus obtained ChLC composite films exhibited dual mechanochromism under ambient temperature.

## Abstract 2

We obtained cellulosic/synthetic polymer composites incorporating a cholesteric liquid crystalline structure by *in situ* polymerization and accomplished dual mechanochromism at room temperature.

## Graphical abstract

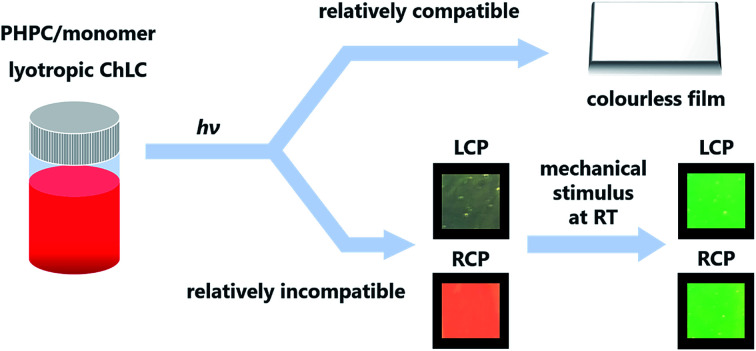

## Introduction

Liquid crystal (LC)/polymer composites are fascinating functional materials that can give excellent mechanical, electrooptical, and thermal properties. Many types of LC/polymer composites have been developed such as polymer-dispersed LCs,^1–3^ LC gel/elastomer,^4–6^ and LC microspheres^7–12^ for application in display devices, sensors, actuators and so on. In particular, cholesteric LCs (ChLCs) have potential for realizing excellent functional materials^13–15^ because they express a selective reflection of circularly polarized light (CPL) which originates from their supramolecular helical structure. In this paper, we focus on the production of ChLC/polymer composites with less well-known optical functionality.

Cellulose is the most abundant natural polymer possessing attractive features involving biocompatibility, biodegradability, and reproducibility so that it is an urgent subject to design functional materials by taking advantage of physicochemical properties of cellulose. Since cellulose derivatives and nanocrystals are capable of forming lyotropic ChLC in concentrated solutions and dispersions, respectively,^16–19^ based on rigidity and chirality, they are useful for preparing ChLC/polymer composites. Researchers have achieved to develop a range of promising composites including electric field-responsive gels,^20–22^ mechanochromic elastomers,^23^ mesoporous photonic materials,^24–26^ and mineralized anisotropic films^27,28^ through combining cellulose derivatives or nanocrystals with synthetic polymers mostly by interpenetrating network (IPN). The IPN formation is carried out to immobilize the ChLC structure in the systems, but it generally leads to dense crosslinking, which may restrict controlling physical properties of the composites.

In our recent work, we prepared ethyl cellulose (EC) concentrated solutions in acrylic acid (AA) monomer and obtained the composites incorporating ChLC structure by *in situ* polymerization of AA without crosslinker.^29^ The product (EC/PAA) films with good mechanical property showed not only wide-ranging colour change but also circular dichroic inversion upon mechanical stimulus.^30^ We called this phenomenon “dual mechanochromism”. EC/PAA films, however, exhibited such mechanochromic behaviours at temperatures higher than 120 °C because the glass transition temperature (*T*_g_) of the composite was ∼120 °C. Although the *T*_g_ of EC composites may be controllable by mixing EC with other monomers that produce polymers with lower *T*_g_, the monomeric solvent allowing EC to form lyotropic ChLC phase has never been reported other than AA so far. In order to accomplish the similar mechanochromism at ambient temperature, therefore, it is preferable to find cellulose derivatives which express ChLC behaviour in various solvents as well as have low *T*_g._

Herein, we prepared propionylated hydroxypropyl cellulose (PHPC) which is flexible at room temperature. We subsequently examined its liquid crystallinity in different monomer solutions and the LC immobilization behaviour of the polymer films obtained by *in situ* photopolymerization of the monomeric solvents. Visual observations and spectroscopies demonstrated that PHPC could express lyotropic ChLC in diverse monomers, but that the mesomorphic structure could be effectively fixed only in certain polymerized products. We analysed the PHPC/synthetic polymer films by wide-angle X-ray diffraction (WAXD), differential scanning calorimetry (DSC), and solid-state NMR and elucidated that a compatibility between PHPC and the coexistent polymer played a key role in immobilizing the ChLC phase in the films. Eventually, we visually and spectroscopically revealed that the resulting PHPC-based ChLC films manifested the dual mechanochromism at ambient temperature.

## Experimental

### Materials

Hydroxypropyl cellulose (HPC) was purchased from Tokyo Chemical Industry Co., Ltd. (TCI) and the molecular parameters are as follows: number- and weight-average molecular weights, 9.9 × 10^4^ and 1.5 × 10^4^ respectively; degree of substitution, DS = 1.4 and MS = 3.9,^28^ where DS and MS denote an average number of substituted hydroxyl group and that of introduced hydroxypropyl group, respectively. Methyl acrylate (MA), methyl methacrylate (MMA), ethyl acrylate (EA), ethyl methacrylate (EMA), isopropyl methacrylate (iPMA), and 4-acryloylmorpholine (ACMO) were obtained from TCI and used after passing through a column packed with activated alumina (Wako Pure Chemical Industries, Ltd. (Wako)). Dehydrated pyridine (Wako), propionic anhydride (TCI), 4-dimethylaminopyridine (DMAP) (Wako), 2-hydroxy-2-methylpropiophenone (HMPPh) (Sigma Aldrich Co. LCC.) were used without further purification.

### Preparation of PHPC

HPC (10 g) and DMAP (1 g) were dissolved in 200 mL of pyridine at 40 °C. 150 mL of propionic anhydride was added into the solution and the reaction was carried out at 40 °C for 24 h. After the reaction, 300 mL of methanol was added to the solution in an ice water bath and it was stirred, followed by dialysis of the mixture in ethanol for 3 days. The dialyzed sample, then, was concentrated by heating on a hot plate thermoregulated at 100 °C and dried in vacuum oven at 40 °C for 24 h to obtain PHPC. The ^1^H NMR measurement (600 MHz) of this sample in CDCl_3_ was performed using ECA 600 (JEOL Ltd.) and obtained signals were assigned as shown in Fig. S1.† DS_Pr_, an average number of introduced propionyl groups per repeating unit of HPC, was calculated from

1 DS_Pr_ = (*I*_c_/2)/{(*I*_ring_ + *I*_a_)/(7 + 3MS)}

where *I*_c_, *I*_ring_, and *I*_a_ are the integrals for the areas of signals c, ring proton, and a, respectively. DS_Pr_ of PHPC used in this work was 3.

### Preparation of PHPC/monomer ChLC solutions

PHPC/monomer ChLC solutions were prepared in a cellulosic concentration range of 76–82 wt% by mixing weighed amounts of PHPC and monomers in vials. In the mixing process, the sample vials were turned upside down and centrifuged (2500 rpm, 10 min) 20 times to accomplish a complete dissolution of PHPC.

### Preparation of PHPC/polymer films

A photoinitiator, HMPPh was added to the PHPC/monomer solutions at a concentration of 0.5 wt% with respect to the monomers and the systems were then mixed by centrifuging with the same manner as preparing the ChLC solutions. The solutions containing HMPPh were spread into filmy shape between two PET films with a 0.3 mm thickness of Teflon spacer. The sandwiched lyotropic samples were placed in a temperature-controlled dark room at 20 °C for 24 h to stabilize the system. The ChLC solutions were subjected to photopolymerization of the monomers at 25 °C by 1 h irradiation of UV light with an intensity maximum at 352 nm.

### Preparation of films composed of pristine synthetic polymers

A photoinitiator, HMPPh was added to the monomers at a concentration of 0.5 wt% and the mixtures were stirred by a magnetic stirrer. The solutions were pored into Teflon Petri dish and exposured to the UV light at 25 °C for 12 h to polymerize them.

### Compression of PHPC/polymer films

PHPC/polymer specimens (10 × 5 × 0.3 mm^3^) cut from the films as-polymerized were used for examination of selective reflection as well as circular dichroism of the films subjected to a compression stress. The samples were compressed at 30 °C using a hot-press apparatus IMC-180C (Imoto Machinery Co.). The applied strain (*ε*) was determined by2
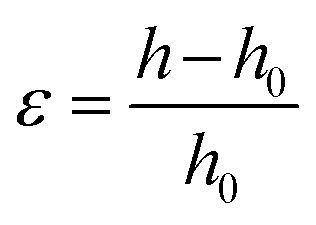
where *h*_0_ and *h* are the thicknesses of the specimens before and after the compression.

### Thermal treatment of PHPC/polymer films

To examine effects of the thermal treatment on the circular dichroism of the PHPC/polymer films, the compressed samples were allowed to heat on a hot-stage regulated at 100 °C for predetermined times.

### Measurements

Selective reflection behavior of the PHPC/monomer solutions and PHPC/polymer films was investigated by visual inspection and spectroscopy. UV-Vis spectra were recorded using a V-550 UV-Vis spectrophotometer (JASCO Co.) over the wavelength range of 300–800 nm with a data interval of 1.0 nm and a scanning rate of 400 nm min^−1^. Circular dichroism (CD) spectra were acquired with a CD spectrometer J-820 (JASCO Co.) over the wavelength range of 300–800 nm with band width of 1.0 nm, sensitivity of 1000 mdeg, and scanning rate of 500 nm min^−1^. The solution samples sandwiched by glass slides with 0.3 mm Teflon spacer were placed where the beam passed through it in the spectrometers. The film specimens put in conventional plastic cells were inserted into the cell holder in the instruments.

Refractive indices of the film samples were measured using an Abbe's refractometer NAR-1T SOLID (ATAGO Co., Ltd.).

Wide-angle X-ray diffraction (WAXD) profile of the film specimens were acquired by Rigaku Ultima-IV diffractometer in a reflection mode with a Nickel-filtered CuKα radiation (0.15418 nm) at 40 kV and 30 mA. The diffraction intensity profiles were collected in the range of 2*θ* = 5–35°.

Differential scanning calorimetry (DSC) was carried out on 10 mg of the film samples using a DSC7020 (Hitachi High-Technologies Corporation). First heating scan was run at a rate of 20°C min^−1^ from −150 °C to 180 °C. After quick cooling to −150 °C following the first heating, second heating scan was done at 20°C min^−1^.

Solid-state NMR measurements were performed at 20 °C with a ECA 500 (JEOL Ltd.) operated at a ^13^C frequency of 125 MHz, using the film samples. The magic angle spinning rate was 7 kHz. ^13^C CP/MAS NMR spectra were measured with a contact time of 2.0 ms, 90° pulse width of 3.3 μs, and 1024 FID signal accumulations. In quantification of proton spin–lattice relaxation times (*T*^H^_1_), a contact time of 1.0 ms was used and wait-time *τ* ranged from 0.1 to 5 s. 512 scans were accumulated for the *T*^H^_1_ measurements.

The morphologies in cross section of the PHPC/polymer films were observed by field-emission scanning electron microscopy (FE-SEM; S-4800, Hitachi High Technologies Co.) using an accelerating voltage of 15.0 kV. The samples were coated with osmium for 20 s using a Neoc-Pro osmium coater (Meiwafosis Co., Ltd.) before observation.

Additional visual inspection was done for the film samples through an eyeglass-type set of left- and right-handed circular polarizers (3D glasses, TY-EP3D20W (Panasonic Co.)) to visualize the effect of mechanical stimulus on circular dichroism of the films.

## Results and discussion

### Selective reflection of PHPC/monomer solutions and PHPC/polymer films

**Fig. 1:**
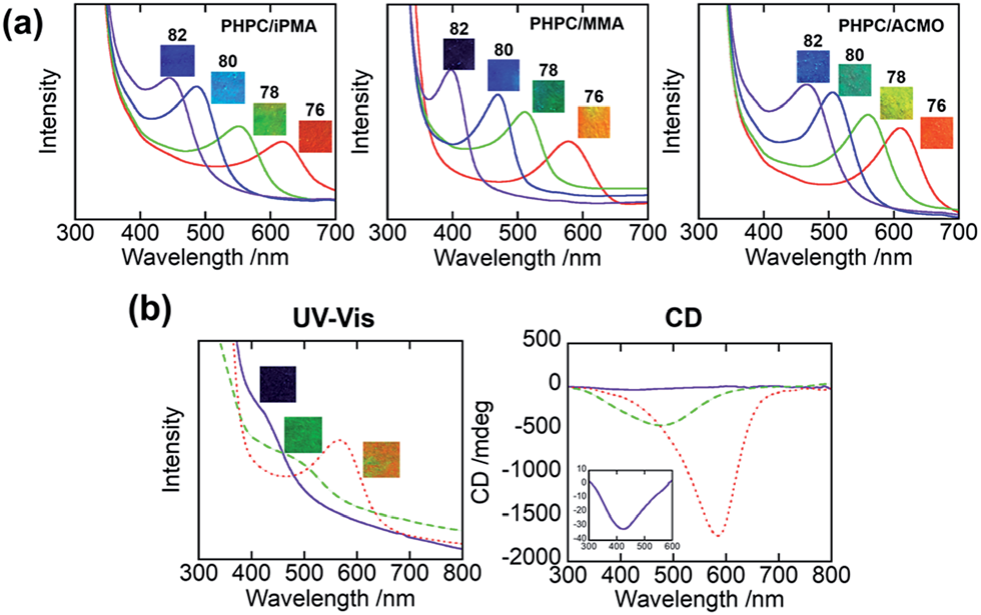
(a) Visual appearances and UV-Vis spectra of PHPC/iPMA, PHPC/MMA, and PHPC/ACMO solutions. Numerals inserted represent the concentrations (wt%) of PHPC in the solutions. (b) Visual appearances, UV-Vis spectra, and CD spectra of 76 wt% PHPC/PiPMA (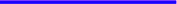), PHPC/PMMA (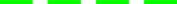), and PHPC/PACMO (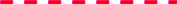) films.

Fig. 1a illustrates visual appearances and UV-Vis spectra for ChLC solutions consisting of PHPC and monomers (isopropyl methacrylate (iPMA), methyl methacrylate (MMA), *N*-acryloylmorpholine (ACMO)) at PHPC contents of 76–82 wt%. All samples showed the selective reflection colour peculiar to ChLC. Similar colouration was also observed when PHPC was dissolved in some other monomers (Fig. S2†). In any of these monomer solvents, the reflection colour of the solutions blue-shifted with an increase in the PHPC-concentration over the visible region. These results indicate that PHPC can form ChLC in various monomeric solvents and exhibit selective reflection of CPL. Incidentally, in the case of unmodified HPC, the option of monomer solvents capable of expressing ChLC phase is poor.

Fig. 1b displays visual appearances, UV-Vis spectra, and circular dichroism (CD) spectra of the PHPC/polymer films obtained by photopolymerization of 76 wt% PHPC ChLC solutions in iPMA, MMA, and ACMO. These retained the visible colours even after the polymerization, while it was impossible with the other monomer systems mentioned in Fig. S2.† As can be seen in the UV-Vis spectra, the reflection wavelengths blue-shifted after the polymerization and the degrees of shifting were in the order of PHPC/PiPMA > PHPC/PMMA > PHPC/PACMO films. The UV absorption intensity was larger for the composites with smaller blue shift. The CD spectra showed a similar blue-shifting and also revealed that the samples with shorter reflection wavelength demonstrated lower reflection intensity suggesting a disruption of the ChLC structure. It was thus found that the efficiency of immobilizing ChLC structure *via* polymerization depended on the sort of the polymerized matrix coexisting.

### WAXD analysis for ChLC structure of PHPC/polymer films

To examine the structural factors disturbing the mesomorphic structure of PHPC/synthetic polymer systems, we discuss the parameters affecting the selective reflection. The selective reflection wavelength (*λ*_max_) in ChLC is related to the cholesteric helical pitch (*P*) and the average refractive index (*ñ*) by the de Vries' equation,^31^

3 *λ*_max_ = *ñP*

so that the decrease in *λ*_max_ by the polymerization is attributable to a reduction of *P* in the systems. In addition, *P* can be described as^30,32^

4 *P* = 360°*d*/*φ*

where *d* is the normal distance in the nematic layers and *φ* is the twist angle defined as an azimuth difference between adjacent nematic layers. The lowering action of *P* can thus be induced by decreasing *d* and/or increase in *φ*.

**Fig. 2:**
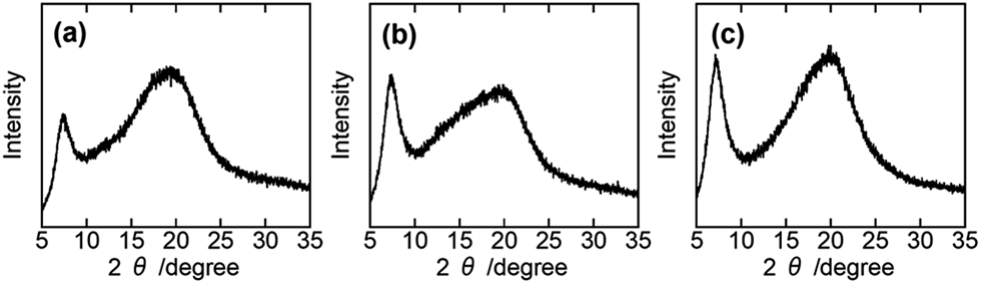
WAXD profiles of 76 wt% (a) PHPC/PiPMA, (b) PHPC/PMMA, and (c) PHPC/PACMO films.

Assuming that short-range order in the cholesteric domain to be analysable in terms of a hexagonal packing of the LC molecules, we can obtain *d* for the samples from their WAXD profiles.^32^Fig. 2 displays WAXD profiles of 76 wt% PHPC/polymer films. We can see a diffraction at 2*θ* = ∼7° assigned to the periodicity for the stacked nematic layers as well as an amorphous halo at 2*θ* = ∼20° in all samples. The set of parameters relating to the selective reflection are listed in Table 1. From these values, the differences of *d* among the films were less than those of *φ*. This indicates that the change in *P* among the polymerized samples was mainly due to the increase in *φ* by the polymerization. In addition, PHPC/PMMA and PHPC/PACMO films which showed relatively intense selective reflection gave strong diffraction arcs in comparison with PHPC/PiPMA film. This means that the molecularly ordered structure of the ChLC phase was considerably maintained in PHPC/PMMA and PHPC/PACMO.

**Table 1:** List of the data relating to the selective reflection for the 76 wt% PHPC/PiPMA, PHPC/PMMA, and PHPC/PACMO films

Samples	*λ*_max_/nm	*ñ*	*P*/nm	*d*/nm	*φ*/°
76 wt% PHPC/PiPMA	433	1.47	295	1.20	1.47
76 wt% PHPC/PMMA	496	1.47	337	1.21	1.30
76 wt% PHPC/PACMO	565	1.49	379	1.22	1.16

### Compatibility test of PHPC/polymer films

**Fig. 3:**
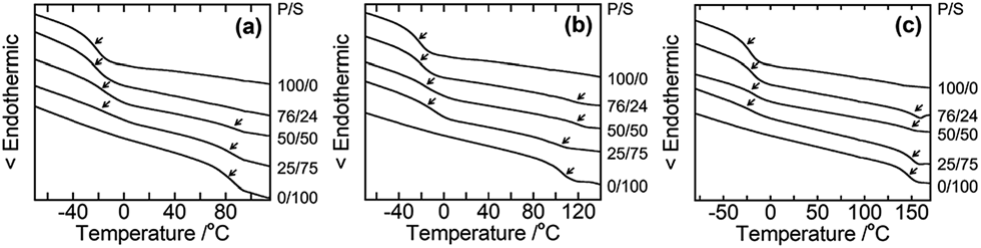
DSC thermograms (2nd heating) of (a) PHPC/PiPMA, (b) PHPC/PMMA, and (c) PHPC/PACMO films at different P/S which is the ratio of PHPC and the synthetic polymers in the composites.

Considering the possibility that the compatibility of PHPC with the synthetic polymers *in situ* formed is related to the efficiency of components in polymer blends is commonly estimated by determination of their *T*_g_. When the constituent polymers are immiscible, the micro-Brownian motion of each polymer occurs independently and separated *T*_g_s can be seen. On the other hand, if a single *T*_g_ appears due to the cooperative micro-Brownian motion of the component polymers, the system can be regarded as miscible on the *T*_g_-detection scale that is usually assumed to be 20–30 nm.^33^Fig. 3 presents DSC thermograms of PHPC/PiPMA, PHPC/PMMA, and PHPC/PACMO films with varied PHPC/synthetic polymer (P/S) ratios. The flexible pristine PHPC (100/0) showed a low *T*_g_ (∼−20 °C). In all composite samples, two *T*_g_s associated with PHPC and the *in situ* synthesized polymers were observed separately regardless of the P/S ratio. Although a single *T*_g_ was observed for 76 wt% PHPC/PiPMA film (denoted as 76/24 in Fig. 3a), this was probably caused by low detection sensitivity of the *T*_g_ for PiPMA component: the single *T*_g_ was at almost same position for that of pristine PHPC. These results revealed that PHPC and these synthesized polymers were immiscible in a scale of 20–30 nm. That is, the mixing state of the constituent polymers in this scale is not the main factor as to whether or not to lock the mesomorphic structure in the composites.

In order to explore the compatibility between PHPC and the polymerized matrix on a larger scale than that investigated by DSC, we performed solid-state NMR measurements on the film samples. It is well established that ^1^H spin–lattice relaxation time in the laboratory frame (*T*^H^_1_) calculated from the solid-state NMR spectra for components of polymer blends provides information on the mixing state of the polymers in a scale of several tens of nanometres. *T*^H^_1_ is a time constant of a relaxation process in which a nuclear spin magnetization perpendicular to the static magnetic fields towards parallel state to the fields. To obtain *T*^H^_1_ practically, we track the intensity of the NMR signal arising from the samples at different times (*τ*) during the relaxation process. We acquired ^13^C CP/MAS NMR spectra of 76 wt% PHPC/PiPMA, PHPC/PMMA, and PHPC/PACMO films and assigned the signals to the corresponding carbons, as shown in Fig. S3.† Because the pristine PHPC was sticky even at room temperature, the solid-state NMR measurement accompanying high-speed rotation was avoided. The relaxation process was monitored on the intensities of the signal f of PHPC and the ones of i, i′, and g′′ + i′′ of PiPMA, PMMA, and PACMO, respectively. *T*^H^_1_ values can be obtained by fitting the carbon resonance intensity to the following exponential equation5
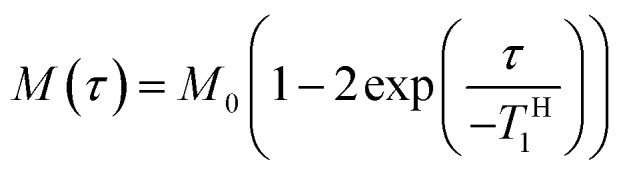
where *M*(*τ*) is the magnetization intensity at a wait-time *τ* and *M*_0_ is the one in an equilibrium state given by *M*(0) = −*M*_0_. By rewriting eqn (5) to (6) and applying a semi-logarithmic plot, we can calculate *T*^H^_1_ from the slope of the obtained straight line.6
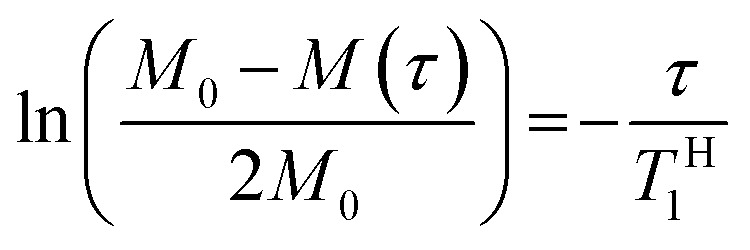


**Fig. 4:**
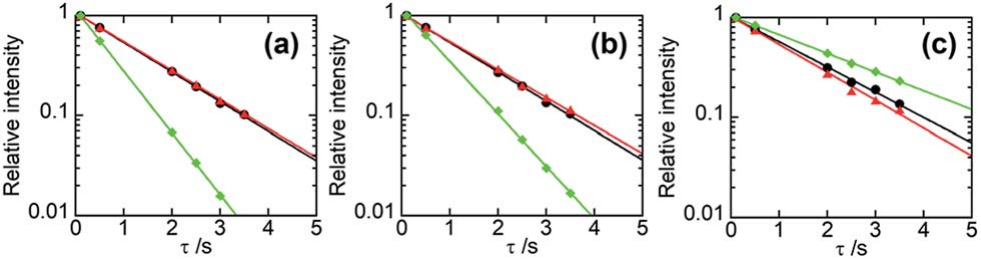
Semi-logarithmic plots of the relative intensities of NMR signals as a function of *τ* for 76 wt% (a) PHPC/PiPMA, (b) PHPC/PMMA, and (c) PHPC/PACMO films. The data of PHPC and the coexisting polymers in the composites are represented in red and black, respectively. The data of the polymers synthesized without PHPC are shown in green. The straight lines were drawn based on eqn (6).

In general, if two polymer components are in a homogeneous mixing state on several tens of nanometres scale, ^1^H spin diffusion can take place, the *T*^H^_1_ values for different protons belonging to the respective components may be equalized. In that case, the straight lines fitted by eqn (6) for those constituents have the same slope and overlap each other. Fig. 4 displays the semi-logarithmic plots of the signal intensity as a function of *τ* for PHPC and the synthesized polymers in the 76 wt% PHPC/PiPMA, PHPC/PMMA, and PHPC/PACMO composite films, together with the data for the corresponding plain synthesized polymers. For PHPC/PiPMA film (Fig. 4a), the regression lines on each component in the composite were almost overlapped and both were apart from the line for the pristine PiPMA. These polymers can be thus considered to be compatible in a scale of *T*^H^_1_ measurements. On the other hand, for the other two composites (PHPC/PMMA (Fig. 4b) and PHPC/PACMO (Fig. 4c), the regression lines of signals of PHPC and polymerized matrix did not overlap. The slopes of the two lines for each combination, however, were apparently different from that of the plain synthesized polymers without PHPC. Namely, although these systems are not completely compatible on the *T*^H^_1_ observation scale, each component can be regarded as being spatially close to each other to the extent affecting the relaxation behaviour. For PHPC/PMMA film, furthermore, two straight lines significantly approached each other in comparison with the case of PHPC/PACMO film. This demonstrates that the compatibility of PHPC with PMMA is higher than that with PACMO.

Table 2 lists *T*^H^_1_s calculated from the slopes of the respective semi-logarithmic plots for PHPC and the *in situ* synthesized polymers in the composites. In the order of PHPC/PiPMA < PHPC/PMMA < PHPC/PACMO, the difference in *T*^H^_1_ between PHPC and the coexisting polymers, that is, the degree of their incompatibility increased. This corresponds to the order of the ability to immobilize the ChLC structure, which was shown in the UV-Vis and CD spectroscopy. Consequently, it was found that the ChLC structure could be more efficiently locked in the films where the constituent polymers were more incompatible in the scale estimated from *T*^H^_1_.

**Table 2:** *T* ^H^_1_ values of PHPC and the synthesized polymers in 76 wt% PHPC/PiPMA, PHPC/PMMA, and PHPC/PACMO films

Samples	*T* ^H^_1_/s	
	PHPC	Synthesized polymers
76 wt% PHPC/PiPMA	1.50	1.47
76 wt% PHPC/PMMA	1.55	1.48
76 wt% PHPC/PACMO	1.56	1.72

These results may be interpreted by considering the synthesized polymers as achiral additives in these ChLC systems. Namely, achiral oligomeric impurities are known to disrupt the cholesteric molecular arrangement, generally making the helical pitch shorter.^34^ In the present PHPC/synthetic polymer systems, achiral polymeric impurities were formed *in situ* during the photopolymerization and they likely disorder the molecular alignment in the mesomorphic structure. This should lead to shortening the helical pitch as well as the blue-shifting the reflection wavelength. The *in situ* synthesized polymers can induce this structural change through affecting the twisting power of the cholesteric helix, as indicated by WAXD measurements. When the formed polymer chains are spatially close to PHPC, the chains can effectively disturb the equilibrium ordered structures of PHPC in the monomers and affect the wavelength and the intensity of the selective reflection. On the basis of this assumption, immobilization of the ChLC structure can be better in incompatible composites. SEM images of 76 wt% PHPC/PiPMA and PHPC/PACMO films (Fig. S4†) may support this hypothesis; the former demonstrated irregular morphologies, while highly ordered texture was observed for the later. If the *in situ* polymerization causes a phase separation with several hundreds nanometres comparable with the wavelength of visible light, however, there is a possibility for another problem such as whitening due to scattering of visible light and reduction in the degree of coloration accompanied therewith. Accordingly, we propose that a moderate compatibility is crucial for fabricating the composites with highly maintained mesomorphic structure.

### Dual mechanochromism of PHPC/PAMO film at room temperature

**Fig. 5:**
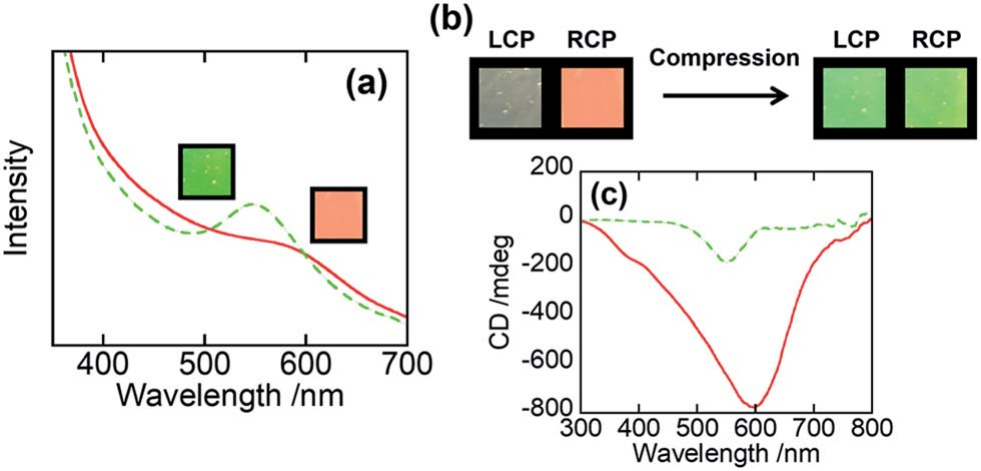
(a) Visual appearances and UV-Vis spectra, (b) visual appearances through circular polarizers, and (c) CD spectra for 76 wt% PHPC/PACMO film before (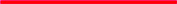) and after (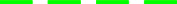) compression at 30 °C. The applied compressive strain and stress were 0.10 and ∼5000 MPa, respectively.

We then examined the mechanochromic property of the PHPC/PACMO system at ambient temperature. Although the *T*_g_ of PACMO (∼150 °C, see Fig. 3c) is much higher than room temperature, a ductility of the moderately compatible (partially miscible) rigid/flexible polymer blend systems can be greater than that of truly miscible ones.^35^Fig. 5a shows visual appearances and UV-Vis spectra of 76 wt% PHPC/PACMO film before and after compression at 30 °C. The applied compressive strain and stress were 0.10 and ∼5000 MPa, respectively. These results clarified that the reflection wavelength of the PHPC/PACMO film blue-shifted in response to the compression. Based on the Lambert–Beer's law, it may be natural to suppose a decrease in the reflection intensity of the film due to a sample thinning by the compression. But contrary to the expectation, the compressed film exhibited a rather stronger selective reflection. The reason of this phenomenon will be investigated in our future work. For the same sample, the effect of the mechanical stimulus on its circular dichroism was examined by visual observation through left and right-handed circular polarizers (LCP and RCP, respectively) (Fig. 5b). Before compression, circular polarized light (CPL) reflected from the sample mainly transmitted through RCP, indicating right-handed circular dichroism. On the other hand, CPL reflected from the compressed film passed through both the LCP and RCP. This means that the circular dichroism of the PHPC/PACMO film was partially inverted by applying the compressive stress. This result was also supported by CD spectra (Fig. 5c). The intensity of the negative signal indicating the reflection of right-handed CPL decreased through compressing the film. This can be attributed to an increase in the reflection of left-handed CPL. Recovering the original right-handed circular dichroism was also achieved by thermal treatment of the compressed sample at 100 °C for 10 s (Fig. S5†). Hence, although the circular dichroism was not fully inverted, the PHPC/PACMO film showed the dual mechanochromism at 30 °C. This cannot be accomplished for the EC/PAA system reported in our previous work. The incomplete inversion is probably because the molecular mobility of PHPC was restricted by the PACMO chains whose molecular motion should be frozen at this temperature.

## Conclusions

We found that PHPC was capable of forming ChLC in a wide range of monomeric solvents. When PHPC/monomer ChLC solutions were subjected to *in situ* photopolymerization, however, the reflection wavelength and intensity of the selective reflection decreased. The degree of decrease in them was dependent on the monomer species. *T*_g_-detection and *T*^H^_1_ measurement elucidated that ChLC structure was more effectively immobilized in the films where PHPC and the coexistent polymer were relatively incompatible. Finally, PHPC/PACMO film exhibited the dual mechanochromism at ambient temperature. The extension of the application temperature of the dual mechanochromism can result in the production of highly functional optical materials.

## Conflicts of interest

There are no conflicts to declare.

## Supplementary Material

RA-008-C8RA04878A-s001
