# Supplementary material for: Exploration of immobilization conditions of cellulosic lyotropic liquid crystals in monomeric solvents by in situ polymerization and achievement of dual mechanochromism at room temperature
Source: RSC Adv. 2018 Jul 10;8(44):24724–30. doi: 10.1039/c8ra04878a (PMC9082404; doi:10.1039/c8ra04878a)
Supplement: RA-008-C8RA04878A-s001 [file RA-008-C8RA04878A-s001.pdf]

Electronic Supplementary Material (ESI) for RSC Advances.
This journal is © The Royal Society of Chemistry 2018

Supporting Information

Exploration of immobilization conditions of cellulosic lyotropic liquid crystal in monomeric solvents by *in-situ* polymerization and achievement of dual mechanochromism at room temperature

Kazuma MIYAGI,[a] Yoshikuni TERAMOTO*,[a,b,c]

[a.] Science of Biological Resources, The United Graduate School of Agricultural Science, Gifu University, Gifu 501-1193, Japan.
[b.] Department of Applied Life Science, Faculty of Applied Biological Sciences, Gifu University, Gifu 501-1193, Japan.
[c.] Center for Highly Advanced Integration of Nano and Life Sciences (G-CHAIN), Gifu University, Gifu 501-1193.

* To whom correspondence should be addressed: teramoto@gifu-u.ac.jp

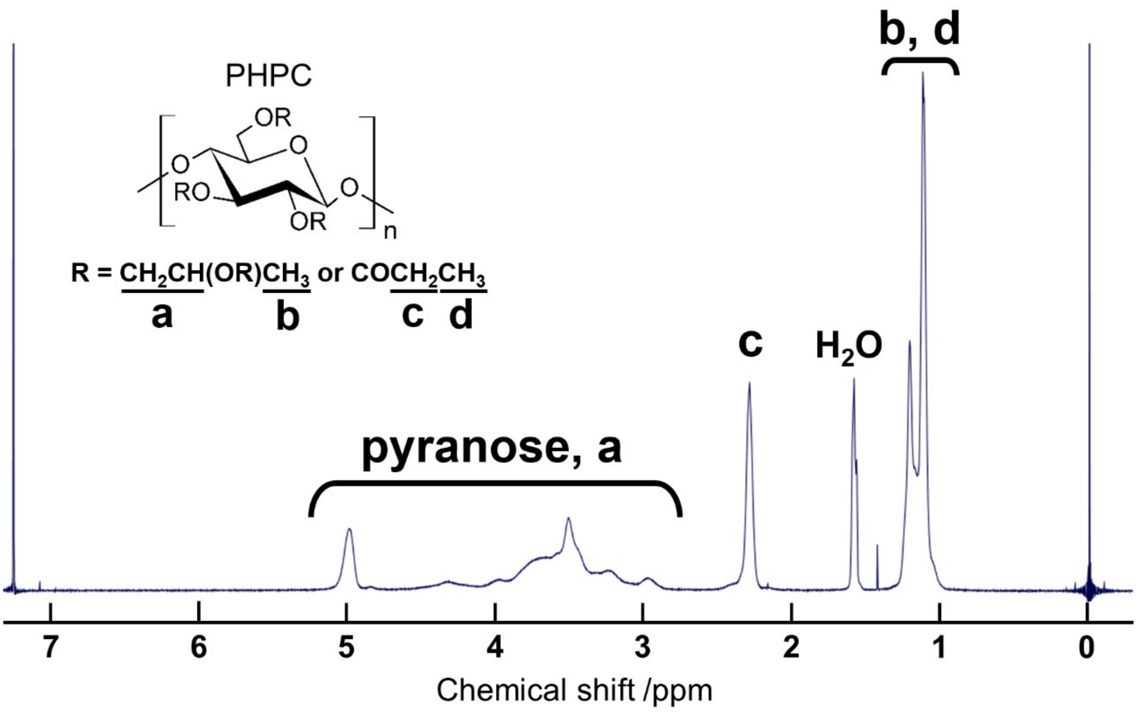

Figure S1 ^{1}H NMR spectrum of PHPC in $CDCl_3$.

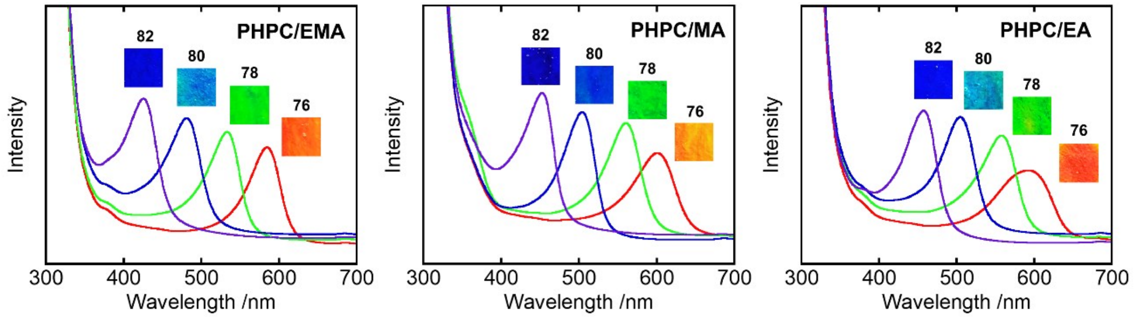

Figure S2 Visual appearances and UV-Vis spectra of PHPC/EMA, PHPC/MA, and PHPC/EA solutions. Numerals inserted represent the concentrations (wt%) of PHPC in the solutions.

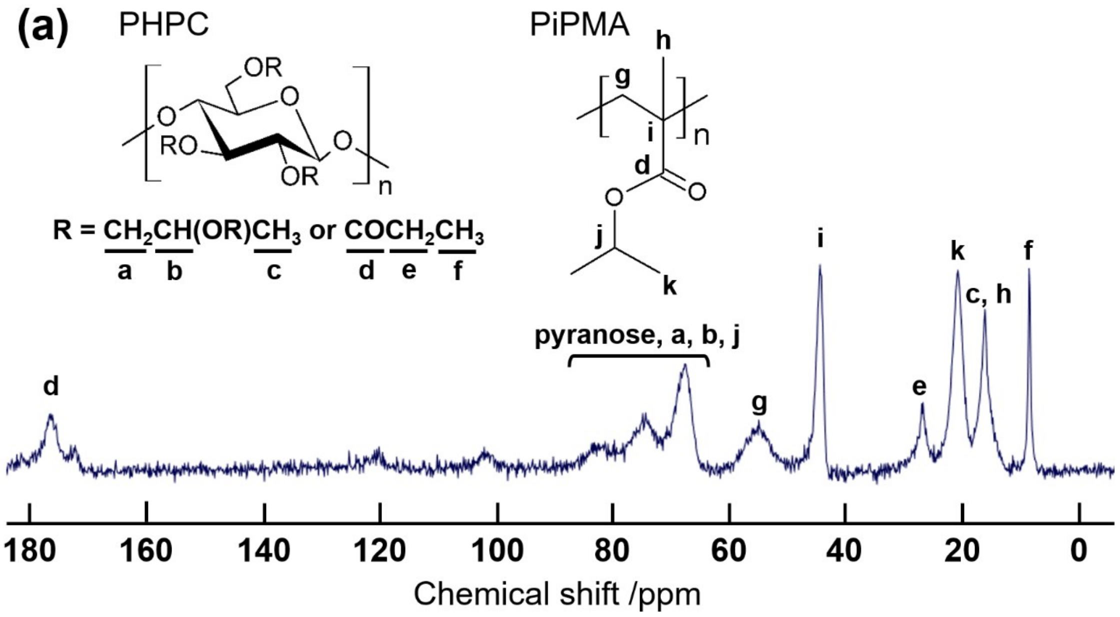

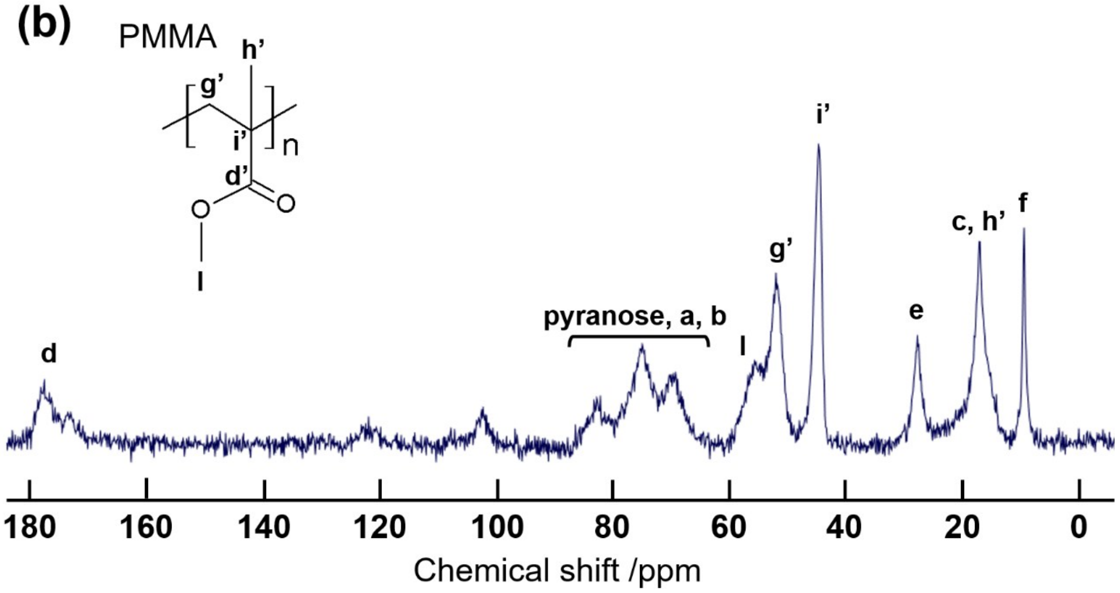

Figure S3 Solid-state ^{13}C CP/MAS NMR spectra of 76-wt% (a) PHPC/PiPMA, (b) PHPC/PMMA, and (c) PHPC/PACMO films.

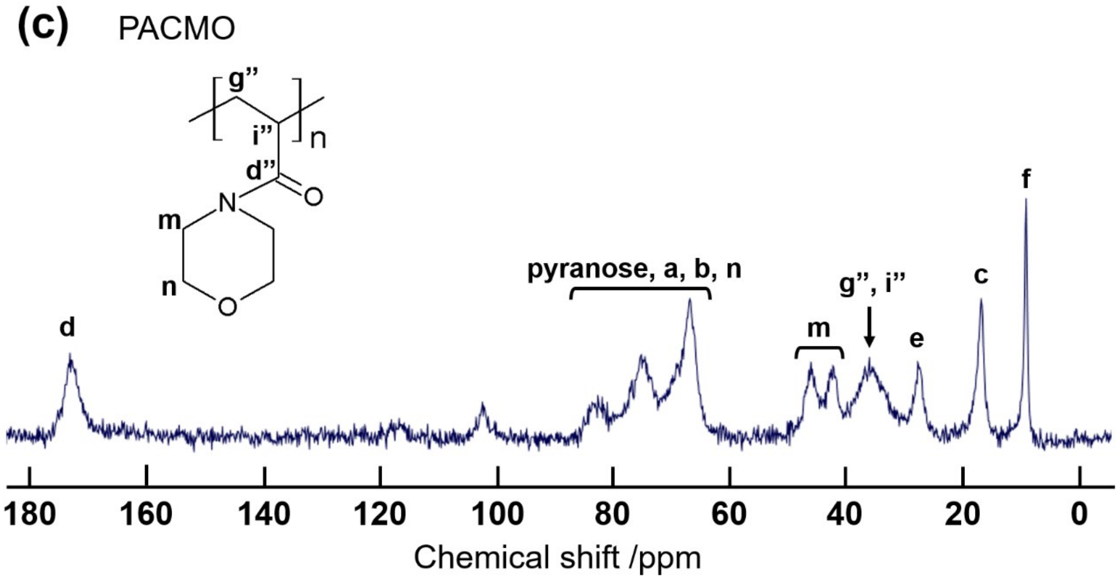

Figure S3 (Continued).

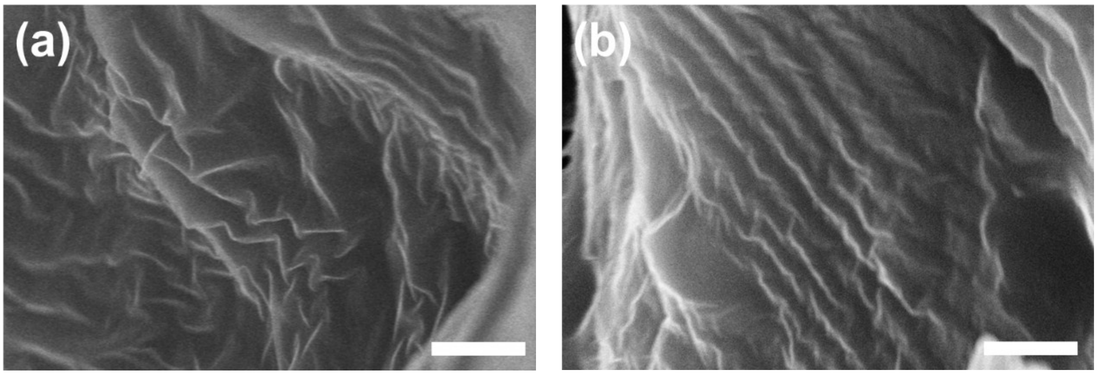

Figure S4 SEM images of fracture surface morphologies of 76 wt% (a) PHPC/PiPMA and (b) PHPC/PACMO films. Scale bar denotes 1 μm.

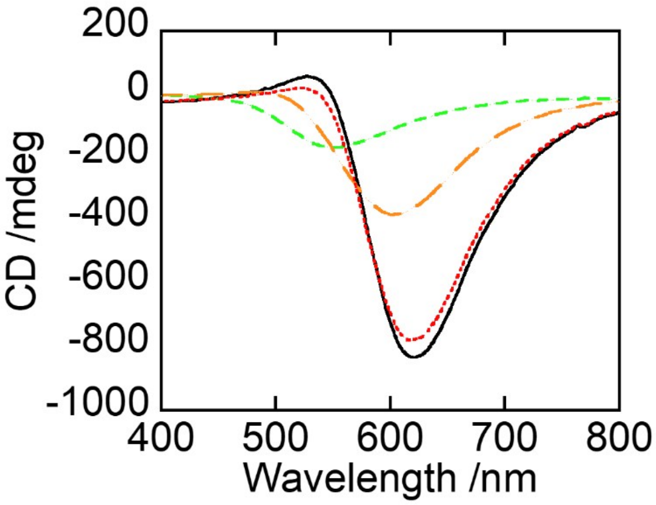

Figure S5 CD spectra of 76 wt% PHPC/PACMO film after the compression at 30°C and subsequent heat treatment at 100°C for 0 s (- - - - -), 5 s (— —), and 10 s (··········) with the spectrum of the as-polymerized one (———).
